# Complete Genome Sequence of Salmonella enterica Serovar Typhimurium Siphophage Siskin

**DOI:** 10.1128/MRA.00188-19

**Published:** 2019-05-02

**Authors:** Chandler O’Leary, Yicheng Xie, Rohit Kongari, Jason J. Gill, Mei Liu

**Affiliations:** a Center for Phage Technology, Texas A&M University, College Station, Texas, USA; Queens College

## Abstract

Bacteriophage Siskin is a member of the χ-like siphovirus phage cluster that infects Salmonella enterica serovar Typhimurium strain LT2. Here, we report the complete 58,476-bp sequence of the Siskin genome, provide confirmation of its genomic termini, and describe a potentially new class of holins and endolysins found in the lysis cassette.

## ANNOUNCEMENT

Salmonella enterica serovar Typhimurium is a foodborne pathogen that causes millions of infections annually (1, 2). The bacteriophage Siskin could be used therapeutically or during food processing to combat this pathogen.

Siskin was isolated in 2016 from wastewater collected in Austin, TX, using S. Typhimurium strain LT2. Host bacteria were cultured on tryptic soy broth or agar (Difco) at 37°C with aeration, and phage were propagated using the soft agar overlay method (3). Phage genomic DNA was prepared using a modified Promega Wizard DNA cleanup kit protocol (4). Pooled indexed DNA libraries were prepared using the Illumina TruSeq Nano LT kit, and a sequence was obtained using the MiSeq V2 500-cycle reagent kit, following manufacturer’s instructions, producing 539,431 reads for the index containing the phage genome. Reads were quality controlled in FastQC (https://www.bioinformatics.babraham.ac.uk/projects/fastqc/), trimmed with FASTX-Toolkit 0.11.6 (http://hannonlab.cshl.edu/fastx_toolkit/download.html), and assembled by SPAdes 3.5.0 (5) into a raw contig at 265.4-fold coverage. Protein-coding genes were predicted using GLIMMER 3.0 (6) and MetaGeneAnnotator 1.0 (7). tRNA genes were predicted with ARAGORN 2.36 (8). Protein functions were predicted primarily by sequence homology using BLASTp 2.2.28 (9), and the conserved domain was searched using InterProScan 5.15-54.0 (10). Rho-independent termination sites were identified via TransTerm (http://transterm.cbcb.umd.edu/). Transmembrane domain and topology were predicted by TMHMM 2.0 (11, 12). All analyses were conducted at default settings through the CPT Galaxy (13) and WebApollo (14) platforms (https://cpt.tamu.edu/).

Out of 74 predicted total proteins of Siskin, 68 are similar to those of phage χ (GenBank accession no. KM458633) by BLASTp, with an E value of <0.001 (15, 16), indicating that Siskin is a member of the χ-like phage cluster (17). Phage Siskin has 92.48% nucleotide similarity to phage Utah (18) (GenBank accession no. KY014601), as determined by progressiveMauve 2.4.0 (19). Like phages χ (15, 16) and Utah (18), Siskin was predicted to package its DNA by a site-specific *cos* mechanism with 5′ extended overhangs. With primers (5′-GGGCGGCTGAGAAAGAATTA-3′ and 5′-CAACCGGGTAAAACCGTAA-3′) facing off the ends of the assembled raw contig, PCR using the ligated genomic DNA was conducted, and the resulting product was Sanger sequenced. The obtained sequence was compared to the direct Sanger resequencing of the phage termini (20), using primer 5′-GGGCGGCTGAGAAAGAATTA-3′ off one end and 5′- CAACCGGGTAAAACCGTAA-3′ off the other end. The Sanger sequencing comparison results confirmed that the 5′ end of the Siskin chromosome is at a position identical to the 5′-overhang sequence determined for phages χ and Utah. Based on the presence of identical terminal sequences (GGTGCGCAGAGC), the Siskin chromosome is predicted to have 12-bp 5′ overhangs with the sequence 5′-GGTGCGCAGAGC-3′, and the phage genome was opened at this point. The complete genome of 58,476 bp has a coding density of 93.91% and a G+C content of 56.55%.

The putative holin of Siskin is predicted to have an N-terminal transmembrane domain with N-out, C-in topology, and the putative endolysin is predicted to have two C-terminal transmembrane domains with N-in, C-in topology. These are novel findings and, if experimentally confirmed, reveal a new class of holins (class IV) and a new class of phage endolysins.

### Data availability.

The genome sequence of phage Siskin was deposited under GenBank accession no. MH631453. The associated BioProject, SRA, and BioSample accession numbers are PRJNA222858, SRR8788536, and SAMN11260697, respectively.
